# The effect of Indigenous American genomic ancestry on type 2 diabetes in Mexico: an analysis of 134 548 individuals from the Mexico City Prospective Study

**DOI:** 10.1016/S2468-2667(25)00305-6

**Published:** 2026-01-27

**Authors:** Jaime Berumen, Pablo Kuri-Morales, Jason M Torres, Elizabeth Barrera, Paulina Baca, Fernando Rivas, Laura Alejandra Ramirez-Tirado, Carlos Gonzalez-Carballo, Alberto Zarza, Georgina Del Vecchyo-Tenorio, Oscar Pérez-Flores, Carlos Pantoja-Melendez, Raúl Ramírez, Diego Aguilar-Ramirez, Louisa Gnatiuc Friedrichs, Jonathan R Emberson, Jesús Alegre-Diaz, Roberto Tapia-Conyer

**Affiliations:** a Unidad de Medicina Experimental, Facultad de Medicina, Universidad Nacional Autónoma de México, Mexico City, Mexico; b División de Posgrado, Facultad de Medicina, Universidad Nacional Autónoma de México, Mexico City, Mexico; c Programa OriGen, Instituto Tecnológico de Monterrey, Monterrey, México; d Clinical Trial Service Unit and Epidemiological Studies Unit, Nuffield Department of Population Health, University of Oxford, Oxford, UK

## Abstract

**Background:**

The prevalence of type 2 diabetes in Mexico is among the highest in the world and a major public health problem. The aim of this study was to examine the association between the percentage of Indigenous American genomic ancestry (AMR) and the prevalence of both prediabetes and type 2 diabetes in a large study of Mexican adults.

**Methods:**

In this cross-sectional study, we analysed data from 134 548 individuals from the Mexico City Prospective Study (MCPS), including sociodemographic, clinical, and genetic data. Type 2 diabetes was defined as a self-reported previous diagnosis, glucose-lowering medication, or glycated hemoglobin (HbA_1c_) of at least 6·5%. Prediabetes was defined as HbA_1c_ between 5·7% and less than 6·5%. Individuals with probable type 1 diabetes were excluded. Logistic regression models were used to estimate associations between higher AMR ancestry percentage and prediabetes and type 2 diabetes, after adjustment for age, sex, and other diabetes risk factors.

**Findings:**

Between April 14, 1998, and Sept 28, 2004, 159 755 participants were recruited into MCPS. Among these, 134 548 participants were selected for subsequent analyses (mean age 52 years [SD 12·6]; 90 688 [67·4%] women and 43 860 [32·6%] men); mean AMR ancestry percentage was 66·2% (SD 17·9). Across AMR tenths (mean AMR ranging from 34·8% [SD 7·9] to 94·7% [SD 2·7]), the prevalence of prediabetes increased from 19·2% (2573 of 13 376) to 26·3% (3529 of 13 421) and of type 2 diabetes from 13·5% (1805 of 13 376) to 23·4% (3142 of 13 421). After adjustment for age and sex, each 20% absolute increase in AMR was associated with substantially increased odds of type 2 diabetes (odds ratio [OR] 1·45 [95% CI 1·43–1·48]) and of prediabetes (OR 1·28 [1·26–1·30]). Further adjustment for socioeconomic status, lifestyle factors, and adiposity reduced these ORs to 1·33 (1·31–1·36) for type 2 diabetes and 1·18 (1·16–1·20) for prediabetes. Consequently, at the population mean AMR ancestry percentage, these fully adjusted ORs were 2·59 (2·44–2·75) for type 2 diabetes and 1·75 (1·65–1·85) for prediabetes, whereas among Indigenous populations with 100% AMR ancestry, ORs were 4·23 (3·85–4·64) for type 2 diabetes and 2·33 (2·14–2·54) for prediabetes. ORs for type 2 diabetes and prediabetes associated with higher AMR were higher for women than for men, for younger than for older participants, and were further reduced in magnitude following additional adjustment for a type 2 diabetes polygenic risk score.

**Interpretation:**

The percentage of inherited AMR ancestry is strongly associated with prediabetes and type 2 diabetes in this admixed Mexican population. These findings suggest that most of the Mexican population has a higher genetic susceptibility to type 2 diabetes than European-ancestry populations, underscoring the need for health systems to implement earlier, more intensive, and population-targeted preventive strategies.

**Funding:**

Mexican Health Ministry, National Council of Science and Technology for Mexico, Wellcome Trust, Cancer Research UK, British Heart Foundation, Kidney Research UK, and UK Medical Research Council.

## Introduction

Type 2 diabetes is a major public health problem in Mexico. According to the National Health Survey, the prevalence of type 2 diabetes in Mexico in 2022 was 18%, approximately twice that reported in non-Hispanic White populations in the USA or in European populations.^1^, ^2^, ^3^ Family history is a well documented risk factor for diabetes, and estimates of the genetic heritability of type 2 diabetes range from approximately 30% to 70% in family-based studies, whereas genome-wide association studies suggest that common genetic variants account for roughly 20% of disease susceptibility.^4^, ^5^, ^6^, ^7^ Given the high proportion of Indigenous American ancestry in the Mexican population (about 63% nationwide^8^ and 66% in Mexico City)^9^ this factor might contribute to the observed high prevalence of type 2 diabetes, as suggested by earlier evidence.^10^ For example, type 2 diabetes prevalence has been found to be much higher in Indigenous American populations than in admixed Latinos,^11^, ^12^ with both populations having higher type 2 diabetes prevalences than populations of European ancestry residing in the USA.^12^, ^13^, ^14^ In addition, the identification of polymorphisms strongly associated with type 2 diabetes in Mexican and Latin American populations within the genes *INS-IGF2*,^15^ *SLC16A11*,^16^ and *HNF1A*,^17^ which are absent or very rare in European populations, supports the hypothesis that Indigenous American genomic ancestry (AMR) could have an important role in the epidemiology of type 2 diabetes in Mexico.

**Research in context**

**Evidence before this study**

We searched the PubMed database for English-language articles containing the terms “Amerindian ancestry” or "Indigenous American ancestry" and “type 2 diabetes” published up to and including 2025. We identified 47 articles, of which only four were case–control studies of type 2 diabetes that assessed genetic ancestry. In these four previously reported case–control studies an association between the proportion of Indigenous American ancestry (AMR) and type 2 diabetes was observed, although the effect sizes varied considerably. In two of these studies, the associations were largely explained by socioeconomic factors, but in both cases the sample size was modest, and the study assessed non-European ancestry rather than AMR ancestry specifically. By contrast, one study involving 16 000 Latino individuals found that the association between AMR ancestry proportion and type 2 diabetes increased after adjustment for socioeconomic factors.

**Added value of this study**

This large study of over 130 000 Mexican adults evaluated the association of Indigenous American ancestry percentage with type 2 diabetes and prediabetes after adjustment for a range of socioeconomic characteristics, lifestyle factors, and markers of adiposity. The findings corroborate some previous studies implicating a direct causal role of AMR ancestry percentage on diabetes while providing clarity about the shape and strength of the associations. The study revealed up to a 20% absolute difference in the estimated prevalence of type 2 diabetes across the full AMR ancestry percentage spectrum (0–100%), reflecting the contrast between populations of predominantly European and unadmixed Indigenous descent in Mexico. The findings also revealed clear differences in the strength of the associations between men and women, possibly due to sex-specific genetic effects or gene–environment interactions that vary with AMR ancestry percentage. Finally, it suggests that at least part of the effect of ancestry on type 2 diabetes is directly attributable to genetic factors, as evidenced by its stronger association among younger individuals and the attenuation of the association after adjustment for a type 2 diabetes-specific polygenic risk score.

**Implications of all the available evidence**

Diabetes is multifactorial and influenced by numerous genetic, environmental, and lifestyle factors. From a public health standpoint, the AMR ancestry percentage seems to have an additional role on top of these factors and might help explain why the prevalence of diabetes in Mexico is even higher than expected solely from the high population mean BMI.

Several studies conducted in mestizo populations from Mexico, other Latin American countries, and Latin American populations residing in the USA have found that the risk of type 2 diabetes increases with the percentage of the genome inherited from Indigenous American ancestral populations.^18^, ^19^ However, other factors that increase the risk of diabetes, such as increased general and central adiposity and lower socioeconomic status, are also more common among those with higher percentage of AMR ancestry.^18^, ^19^, ^20^ Most previous studies examining AMR ancestry and type 2 diabetes have had a relatively small sample size and adjusted only for a limited range of potential confounders.^11^, ^12^ In addition, most studies have estimated the effect of ancestry on type 2 diabetes by contrasting individuals with 0% versus 100% ancestry, rather than focusing on the mean AMR ancestry proportion of a population, which is more relevant from a public health perspective. Furthermore, no previous studies of Latin American populations have investigated the effect of AMR on prediabetes, have included undiagnosed type 2 diabetes, or have explored whether any AMR-associated risk for type 2 diabetes differs by sex and age.

The aims of the current study were to investigate the association between the percentage of AMR ancestry and the prevalence of prediabetes and type 2 diabetes (including undiagnosed and previously diagnosed type 2 diabetes) among participants in the Mexico City Prospective Study (MCPS). Secondary aims were to assess whether associations vary by age and sex, and to estimate the extent to which they were explained by socioeconomic and lifestyle factors, markers of adiposity, and a polygenic risk score for type 2 diabetes.

## Methods

### Study design and participants

Participants in this cross-sectional study were recruited into the MCPS between April 14, 1998, and Sept 28, 2004.^21^ MCPS includes more than 150 000 individuals aged 35 years or older from the municipalities of Iztapalapa and Coyoacán of Mexico City. During recruitment, a survey was conducted, which collected self-reported sociodemographic data (ethnicity data were not collected), lifestyle information, medical history, and medication use. In addition, physical measurements were taken and a blood sample was collected. Ethical approval was obtained from the Mexican Ministry of Health, the Mexican National Council of Science and Technology (reference 0595P-M), and the University of Oxford (reference C99.260). All participants provided written informed consent.

### Definition of diabetes and prediabetes

Previously diagnosed diabetes was defined as self-reported previous medical diagnosis at recruitment or use of any glucose-lowering medication. Among individuals without previously diagnosed diabetes, undiagnosed diabetes was defined as glycosylated haemoglobin (HbA_1c_) of at least 6·5% (≥48 mmol/mol) and prediabetes was defined as HbA_1c_ of at least 5·7 mmol/mol up to but not including 6·5%. Those with previously diagnosed diabetes who reported insulin intake and who had an estimated age at onset younger than 35 years were considered likely to have type 1 diabetes.

### Genetic ancestry analysis

As described previously,^9^ the Admixture program (version 1.3.0) was used to determine ancestry proportions in 138 511 individuals from the study, based on genotyping with 650 000 markers using the GSA version 2.0 chip (Illumina, San Diego, CA, USA). A reference dataset of 3964 samples representing African, American, East Asian, and European ancestries was used. This dataset included 765 samples of African ancestry from the 1000 Genomes Project (n=661) and the Human Genome Diversity Project (HGDP; n=104), 658 samples of European ancestry from the 1000 Genomes Project (n=503) and HGDP (n=155), 727 samples of East Asian ancestry from the 1000 Genomes Project (n=504) and HGDP (n=223), and 1814 American samples. The American samples included 716 Indigenous Mexican samples from the Metabolic Analysis of an Indigenous Sample study, 64 Mexican American admixed samples from Los Angeles, 21 Maya samples and 13 Pima samples from HGDP, and 1000 unrelated Mexican samples from the MCPS.

### Statistical analysis

The analysis in the current report excluded individuals older than 85 years at recruitment, those considered likely to have type 1 diabetes, those with unknown HbA_1c_ concentrations, and those for whom genotyping data were not available for estimating ancestry. Among the remaining individuals, the prevalence of prediabetes and type 2 diabetes (undiagnosed or previously diagnosed) was first assessed visually across percentiles of the AMR ancestry distribution (ie, across 100 equally sized groups on the basis of the distribution of the AMR ancestry percentage). This method was done both overall and separately for men and women. Logistic regression was then used to estimate odds ratios (ORs) for prediabetes and type 2 diabetes associated with increased AMR ancestry percentage, initially considering AMR ancestry percentage across ten equally sized categories (ie, from the bottom to the top tenth of the distribution, with the reference group being participants in the bottom tenth) and, subsequently, considering AMR ancestry percentage as a continuous variable (with the log OR and its SE scaled to correspond to a 20% absolute increase in AMR ancestry). These logistic regression models were initially adjusted for age and sex (minimal adjustment), then further adjusted for other diabetes risk factors, which could plausibly lie on a causal pathway between AMR ancestry percentage and diabetes: socioeconomic factors (district, educational level, and income); lifestyle factors (smoking, alcohol consumption, fruit and vegetable consumption, type of cooking oil used, and physical activity); and markers of adiposity (BMI and waist-to-hip ratio). These other risk factors were included in the model as either continuous variables or as categorical variables (table). Analyses were done in the whole cohort and separately for men and women.

**Table:** Baseline characteristics of the 134 548 included participants

			**Women (N=90 688)**	**Men (N=43 860)**	**Total (N=134 548)**
Age, years			51·6 (12·5)	52·9 (12·8)	52 (12·6)
Adiposity					
	BMI, kg/m^2^		29·6 (5·3)	28·4 (4·3)	29·1 (5·1)
	Waist circumference, cm		93·5 (12·2)	96·5 (10·6)	94·5 (11·8)
	Hip circumference, cm		106·4 (11·4)	101·1 (8·2)	104·7 (10·8)
	Waist-to-hip ratio		0·88 (0·07)	0·95 (0·07)	0·9 (0·08)
Socioeconomic and lifestyle factors					
	Income, pesos		879 (2210)*	3310 (5216)	1672 (3669)
	Educational level				
		University or high school	10 175 (11·2%)	10 193 (23·2%)	20 368 (15·1%)
		Middle school	21 680 (23·9%)	11 407 (26%)	33 087 (24·6%)
		Elementary school	45 418 (50·1%)	18 313 (41·8%)	63 731 (47·4%)
		Other	13 415 (14·8%)	3947 (9·0%)	17 362 (12·9%)
Tobacco use					
	Never		56 786 (62·6%)	8873 (20·2%)	65 659 (48·8%)
	Former		15 920 (17·6%)	15 942 (36·3%)	31 862 (23·7%)
	Current		17 982 (19·8%)	19 045 (43·4%)	37 027 (27·5%)
Alcohol use					
	Never		24 014 (26·5%)	2744 (6·3%)	26 758 (19·9%)
	Former		10 851 (12·0%)	7862 (17·9%)	18 713 (13·9%)
	Current		55 823 (61·6%)	33 254 (75·8%)	89 077 (66·2%)
Regular leisure-time physical activity					
	None		73 853 (81·4%)	30 783 (70·2%)	104 636 (77·8%)
	At least 1 day a week		16 835 (18·6%)	13 077 (29·8%)	29 912 (22·2%)
Fruit and vegetable consumption					
	Never		658 (0·7%)	770 (1·8%)	1428 (1·1%)
	1–2 per week		13 566 (15·0%)	9500 (21·7%)	23 066 (17·1%)
	3–4 per week		24 787 (27·3%)	13 128 (29·9%)	37 915 (28·2%)
	5–7 per week		51 677 (57·0%)	20 462 (46·7%)	72 139 (53·6%)
Fried food consumption					
	Never		11 099 (12·2%)	4595 (10·5%)	15 694 (11·7%)
	1–2 per week		53 815 (59·3%)	22 084 (50·4%)	75 899 (56·4%)
	3–4 per week		15 252 (16·8%)	8983 (20·5%)	24 235 (18%)
	5–7 per week		10 522 (11·6%)	8198 (18·7%)	18 720 (13·9%)
Diabetes					
	No prediabetes or diabetes		53 106 (58·6%)	26 179 (59·7%)	79 285 (58·9%)
	Prediabetes		20 663 (22·8%)	9348 (21·3%)	30 011 (22·3%)
	Type 2 diabetes		16 919 (18·7%)	8333 (19·0%)	25 252 (18·8%)
Ancestry proportion					
	Indigenous American		0·664 (0·178)	0·659 (0·180)	0·662 (0·179)
	European		0·29 (0·16)	0·296 (0·162)	0·292 (0·161)
	African		0·037 (0·028)	0·037 (0·028)	0·037 (0·028)
	East Asian		0·008 (0·016)	0·009 (0·019)	0·008 (0·017)

Data are n (%) or mean (SD).

* Nearly two-thirds of women reported not having a salary (75% of them were married or living with a partner).

Using the fully adjusted logistic regression models, the predicted prevalence of prediabetes and type 2 diabetes was then calculated for the extreme ends of the AMR ancestry distribution (ie, 0% and 100%). Prevalence was derived using the equation *p*=*A*/(1 + *A*), where *A* was the model-predicted odds of the outcome at the specified level of AMR. Because the term *A* in the equation includes the model intercept term, *p* can be thought of as reflecting the model-predicted value at the average values of all other covariates included in the model.

Sensitivity analyses include reporting results separately by age at recruitment (<50 *vs* ≥50 years) and inclusion into the fully adjusted model of a polygenic risk score for type 2 diabetes derived from a transancestry type 2 diabetes genome-wide association study meta-analysis by the type 2 diabetes Global Genomics Initiative.^22^ Statistical analyses were done using SPSS version 30.

### Role of the funding source

The funders of the study had no role in data collection, data analysis, data interpretation, writing of the manuscript, or the decision to submit.

## Results

Between April 14, 1998, and Sept 28, 2004, 159 755 participants were recruited into MCPS. Of these, 154 697 (96·8%) had information on medical history and medication use, and had HbA_1c_ measured. Of these, 251 (0·2%) were classified as likely to have type 1 diabetes and were excluded. Genetic data allowing estimation of the percentage of inherited Indigenous American ancestry were available for 137 849 (89·1%) of these individuals. Of these, 3301 (2·4%) had missing data for covariates or were older than 85 years at recruitment and were excluded, leaving 134 548 individuals in the subsequent analyses.

The mean age of the 134 548 participants was 52 years (SD 12·6), with 90 688 (67·4%) women and 43 860 (32·6%) men (table). 25 252 (18·8%) participants had type 2 diabetes, and 30 011 (22·3%) had prediabetes. Mean BMI was higher in women than men but mean waist-to-hip ratio was lower in women than men. 19 045 (43·4%) men and 17 982 (19·8%) women reported smoking cigarettes, 33 254 (75·8%) men and 55 823 (61·6%) women reported drinking alcohol, and 30 783 (70·2%) men and 73 853 (81·4%) women reported doing no regular leisure-time physical activity. 10 193 (23·2%) men and 10 175 (11·2%) women were educated to at least high-school level. The mean percentage of inherited AMR ancestry in the genome was 66·2% (SD 7·9) for the whole sample (65·9% [18·0] in men and 66·4% [17·8] in women). When subdivided into tenths of the AMR ancestry percentage distribution, the mean AMR ancestry percentage in those in the lowest tenth was 34·8% (SD 7·9) and in the highest tenth was 94·7% (2·7) across the whole sample, with very similar percentages in both sexes. The baseline characteristics of the men and women by tenth of AMR ancestry percentage are shown in the appendix (pp 2–3).

**Figure 1:**
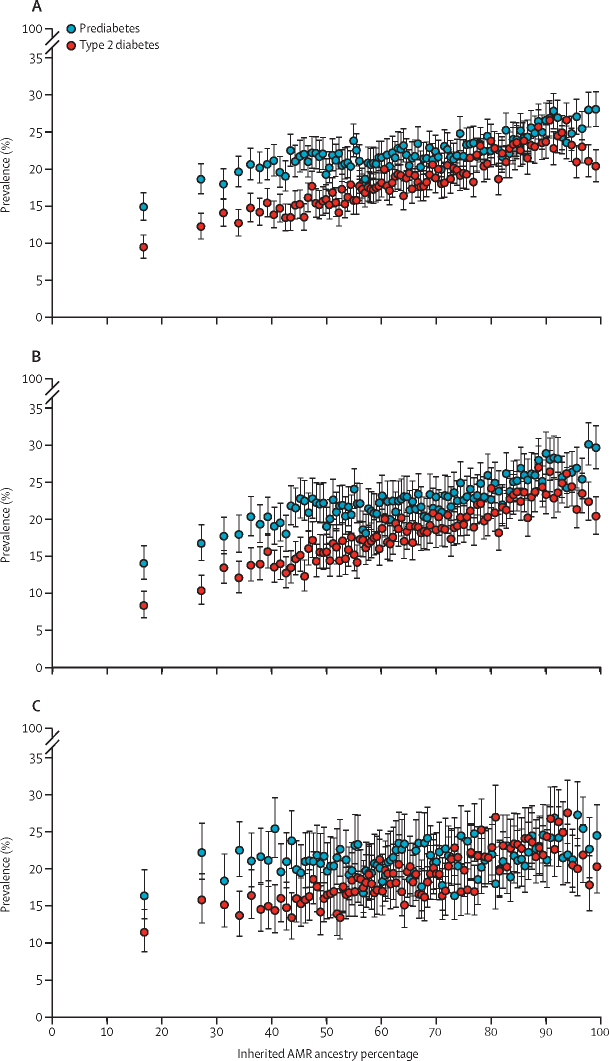
Prevalence of prediabetes and type 2 diabetes by percentiles of inherited AMR ancestry percentage, overall and separately by sex The unadjusted prevalences of prediabetes and type 2 diabetes by percentiles of AMR ancestry proportion are shown for the overall sample (A), women (B) and men (C). AMR=Indigenous American ancestry.

**Figure 2:**
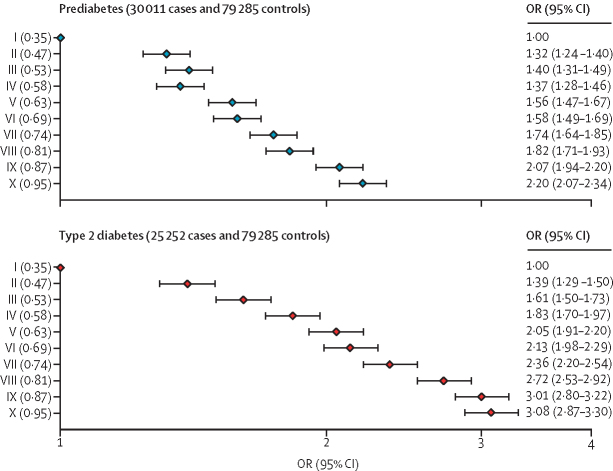
ORs for prediabetes and type 2 diabetes by tenths of the inherited AMR ancestry percentage ORs for prediabetes (upper panel) and type 2 diabetes (lower panel) are presented by tenths of the AMR ancestry adjusted for age and sex. Mean AMR percentage in each group is shown in parentheses for each of the ten groups. The horizontal line through each point represents the 95% CI for that tenth relative to individuals in the bottom tenth (reference group). AMR=Indigenous American ancestry. OR=odds ratio.

There were clear positive associations between the AMR ancestry percentage and the prevalence of both prediabetes and type 2 diabetes across the full AMR ancestry percentage distribution (figure 1). This finding was particularly clear for women. Between the bottom and top tenth of the AMR ancestry percentage distribution, the observed prevalence of prediabetes for men and women combined increased from 2573 (19·2%) of 13 376 individuals to 3529 (26·3%) of 13 421 and the observed prevalence of type 2 diabetes increased from 1805 (13·5%) of 13 376 individuals to 3142 (23·4%) of 13 421. These estimates were broadly similar for men and women (appendix pp 2–3). After adjustment for age and sex, the log odds of prediabetes and of type 2 diabetes increased fairly linearly across tenths of the AMR ancestry percentage distribution (figure 2). Compared with those in the bottom tenth of the AMR ancestry percentage distribution, those in the top tenth had an age-adjusted and sex-adjusted OR of 2·20 (95% CI 2·07–2·34) for prediabetes and 3·08 (2·87–3·30) for type 2 diabetes.

**Figure 3:**
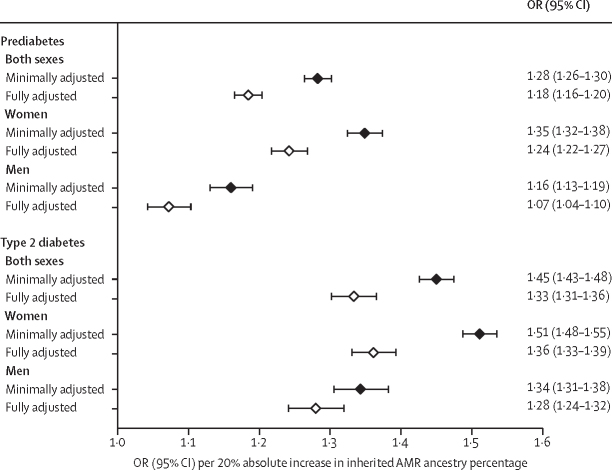
ORs for prediabetes and type 2 diabetes per 20% absolute increase in the inherited AMR ancestry percentage, overall and separately by sex and level of adjustment Minimal adjustment includes adjustment for age and sex (or just age for the sex-specific results). Full adjustment also includes socioeconomic factors, lifestyle factors, BMI, and waist-to-hip ratio. Horizontal lines reflect 95% CIs. AMR=Indigenous American ancestry. OR=odds ratio.

In the minimally adjusted model, each 20% absolute increase in the AMR ancestry percentage was associated with a 28% increase in the odds of prediabetes and a 45% increase in the odds of type 2 diabetes (figure 3). These ORs were larger for women than for men; for example, for women, the OR for prediabetes associated with a 20% absolute increase in AMR ancestry percentage was 1·35 (95% CI 1·32–1·38), whereas for men, it was 1·16 (1·13–1·19), and for type 2 diabetes the OR was 1·51 (1·48–1·55) for women and 1·34 (1·31–1·38) for men.

In both men and women, further adjustment for socioeconomic factors, lifestyle factors, and markers of adiposity (ie, the fully adjusted model) reduced the magnitude of the log ORs associated with AMR ancestry percentage by between a quarter and a third (figure 3). However, even after full adjustment, each 20% absolute increase in the AMR ancestry percentage was still associated with an 18% increase in the odds of prediabetes and a 33% increase in the odds of type 2 diabetes. In women, these ORs were 1·24 (1·22–1·27) for prediabetes and 1·36 (1·33–1·39) for type 2 diabetes, whereas in men they were 1·07 (1·04–1·10) for prediabetes and 1·28 (1·24–1·32) for type 2 diabetes.

Rescaling these fully adjusted ORs to correspond to the population mean AMR ancestry percentage of 66%, the odds ratios would be 2·05 (95% CI 1·91–2·19) for prediabetes and 2·77 (2·56–2.99) for type 2 diabetes in women, and 1·26 (1·14–1·39) for prediabetes and 2·26 (2·04–2·50) for type 2 diabetes in men. With further extrapolation to correspond to Indigenous populations with 100% AMR ancestry, the ORs would be 2·96 (2·66–3·29) for prediabetes and 4·68 (4·16–5·26) for type 2 diabetes in women, and 1·42 (1·23–1·64) for prediabetes and 3·44 (2·94–4·01) for type 2 diabetes in men. Transforming the model-predicted odds of prediabetes and of type 2 diabetes into model-predicted prevalences, the overall predicted prevalence of prediabetes (in men and women combined) increased from 14% for those with no AMR ancestry percentage to 28% for those with 100% AMR ancestry, whereas the predicted prevalence of type 2 diabetes increased from 8% at zero AMR ancestry percentage to 28% for those with 100% AMR ancestry.

In sensitivity analyses, we repeated the main analyses separately for those younger than 50 years at recruitment and those aged at least 50 years (appendix p 4). For both prediabetes and type 2 diabetes, ORs associated with an increase in the AMR ancestry percentage were somewhat larger for those younger than 50 years at recruitment than for those aged at least 50 years. For example, in the minimally adjusted model, the OR for prediabetes associated with a 20% increase in the AMR ancestry percentage was 1·36 (1·33–1·39) in those younger than 50 years and 1·25 (1·22–1·27) in those aged 50 years or older, whereas the OR for type 2 diabetes was 1·52 (95% CI 1·48–1·57) in those younger than 50 years and 1·43 (1·40–1·46) in those aged at least 50 years. For both outcomes, these age-specific differences were somewhat reduced on full adjustment.

Finally, we added into the fully adjusted model a polygenic risk score for type 2 diabetes derived from a transancestry type 2 diabetes GWAS meta-analysis by the type 2 diabetes Global Genomics Initiative (appendix p 5). The correlation between the AMR ancestry percentage and the type 2 diabetes genetic risk score (GRS) was 0·12. Addition of the type 2 diabetes GRS into the fully adjusted models further reduced the OR for prediabetes and type 2 diabetes associated with higher AMR ancestry percentage. For prediabetes, the OR for each 20% absolute increase in the AMR ancestry percentage reduced to 1·15 (95% CI 1·13–1·17) upon inclusion of the type 2 diabetes GRS, whereas for type 2 diabetes it reduced to 1·26 (1·23–1·28).

## Discussion

In this study, we found that the odds of both type 2 diabetes and prediabetes increased steadily with higher AMR ancestry percentage. For both outcomes, odds ratios were larger for women than men, and although up to a third of the magnitude of these associations was explained by adiposity and other factors (some of which might plausibly lie along a causal pathway), the associations remained substantial even after such adjustment. Indeed, on the basis of the fully adjusted models, the predicted prevalence of prediabetes across all participants approximately doubled (from 14% to 28%) between estimated AMR ancestry percentages of 0% and 100%, whereas the predicted prevalence of type 2 diabetes more than trebled (from 8% to 28%).

The association between AMR ancestry percentage and type 2 diabetes observed in this study is stronger than that reported in a previous case–control study of 4662 individuals from the Mexican American Cohort study, in which each 10% higher Native American ancestry percentage was associated with a 7·7% increase in the odds of diabetes after controlling for BMI (hence, a 20% increase in Native American ancestry was associated with a 16% increase in odds).^18^ An analysis from the All of Us study,^20^ which included 16 966 participants who self-identified as Hispanic, found more than twice higher odds of type 2 diabetes among Hispanics than in those who self-identified as European,^20^ but only an OR of 1·15 for type 2 diabetes when comparing individuals with 100% versus 0% Native American ancestry. In our study, adjusting for socioeconomic status reduced the magnitude of the association between AMR and type 2 diabetes by about a third. In a case–control study of 931 individuals from Mexico and Colombia, socioeconomic status explained about two-thirds of the association between non-European ancestry and type 2 diabetes,^12^ whereas in another case–control study of 1275 individuals in Colombia the association between ancestry and type 2 diabetes was no longer significant after adjustment for socioeconomic status.^19^ In the previously mentioned analysis from All of Us,^20^ the relationship between AMR ancestry and type 2 diabetes was weaker among those with lower socioeconomic status than among those with higher socioeconomic status. However, that study relied on electronic health records to identify type 2 diabetes cases, which might have underestimated diagnoses among individuals with lower socioeconomic status or greater social deprivation. In addition, it did not adjust for lifestyle factors or adiposity markers, leaving open the possibility that the observed amplification was due to other risk factors. Our finding of greater effects of AMR ancestry on the odds of prediabetes and type 2 diabetes in women than men has not, to our knowledge, previously been reported.

We also found that the ORs for prediabetes and type 2 diabetes associated with a higher AMR ancestry percentage were larger for younger than older participants, and were reduced in magnitude after further adjustment for a polygenic risk score for type 2 diabetes. Together, these observations suggest that the effect of genomic ancestry on type 2 diabetes has a direct genetic component. Although a few type 2 diabetes-associated variants are more frequent among Indigenous or admixed American populations (eg, risk alleles at the *SLC16A11*, *INS–IGF2*, and *HNF1A* loci), there are probably many others yet to be discovered.^15^, ^16^, ^17^ Any direct effects of AMR ancestry on type 2 diabetes might reflect the aggregate of many locus-specific effects rather than any single variant. Although some variants with higher AMR allele frequencies exert protective effects (such as the minor allele of rs149483638 at the *INS–IGF2* locus),^15^ these are probably counterbalanced by a greater number of risk-increasing alleles or alleles with larger positive effect sizes and higher frequencies (eg, variants in *SLC16A11*) among individuals with a greater percentage of AMR ancestry.

We do not know when these genetic differences began to accumulate in populations with Indigenous American ancestry. However, some variants (eg, missense variants at *SLC16A11*) derive from archaic introgression, whereas others—such as the R230C variant at *ABCA1*—might have arisen or increased in frequency in the Americas under positive selection.^16^, ^23^ However, interaction with environmental factors linked to type 2 diabetes—most notably obesity and high intake of refined carbohydrates and sugar-sweetened beverages—might have an additional role.^24^ In Mexico, although genetic variation associated with Indigenous American ancestry has been shaped over thousands of years, the diabetes epidemic emerged only recently. The prevalence of type 2 diabetes accelerated in the 1990s and became fully apparent in the early 2000s, in parallel with a sustained rise in obesity, which became widespread in the mid-20th century and expanded markedly from the 1970s onward.^25^, ^26^ The marked difference that persists between men and women in the association between AMR and type 2 diabetes—even after adjusting for all relevant factors—also suggests the possible existence of sex-specific genetic effects or variants that respond differentially to hormonal stimuli in males and females.^27^, ^28^

The key strength of this study is its large sample size, which allowed for separate analyses to be done reliably among both men and women. We were able to adjust for a range of other risk factors for diabetes (which could act as mediators of any relationships between AMR ancestry proportion and type 2 diabetes), but did not have information on important dietary exposures such as sugar-sweetened beverages or refined carbohydrates.^29^ Similarly, AMR ancestry is strongly associated with socioeconomic factors and although we adjusted for district, educational level, and income, it is possible that other pertinent and complex social aspects of genetic ancestry remain unaccounted for in our analysis. Diabetes was ascertained through a combination of self-reported diagnoses, diabetes medications, and measured HbA_1c_, and therefore should be reliable. The availability of HbA_1c_ also meant that we were able to estimate associations not just with type 2 diabetes but also with prediabetes. However, type 1 diabetes was inferred on the basis of approximate age at diagnosis and use of insulin. Participants were recruited from just two districts of Mexico City and so are not representative of the whole of Mexico (or even the whole of Mexico City). However, studies of non-representative cohorts of individuals can provide reliable evidence about the associations of risk factors with disease that are widely generalisable.^30^, ^31^ Participants varied considerably in their estimated AMR ancestry percentage (from 17% in the bottom percentile of the distribution to 99% in the top percentile), so the relevance of AMR to type 2 diabetes could be evaluated reliably across this range. Nonetheless, this result does mean that the predicted prevalence at 0% AMR ancestry reflects an extrapolation from the data. We did not examine the age of type 2 diabetes diagnosis for those with previously diagnosed type 2 diabetes and so did not explore whether the influence of AMR on type 2 diabetes might vary with age at onset, although we did see larger ORs for younger participants than for older participants. Consistent with most prospective studies, variables included in the models were either self-reported or directly measured, although it should be acknowledged that information on socioeconomic status was scarce. Finally, the ancestry proportion estimates used in this study were generated from a single method (ie, ADMIXTURE). However, previous work has shown that these estimates are consistent with those obtained from RFMix—a haplotype-based procedure for estimating local ancestry.^9^

Our findings highlight the importance of considering Indigenous American ancestry in understanding the burden of type 2 diabetes in admixed Latin American populations. However, the magnitude (and perhaps even the direction) of the association between AMR genomic ancestry and type 2 diabetes could vary across regions because allele frequencies, linkage disequilibrium, gene–environment interactions, lifestyle patterns, and social factors could differ markedly between communities.^32^ Beyond the observed association, several key questions arise, which we are currently exploring. These questions include identifying the complete set of genetic variants within Indigenous American ancestries (ie, the AMR variome) and ancestry-specific variants associated with type 2 diabetes, the potential role of AMR in the progression from prediabetes to type 2 diabetes, and whether AMR contributes directly to the excess mortality associated with type 2 diabetes in this population.

In summary, in this large study of Mexican adults, estimated percentage of AMR ancestry was associated with more than a trebling in the prevalence of type 2 diabetes across its full range. Diabetes is a multifactorial disease influenced by a wide range of genetic, environmental, and lifestyle factors. In this study of individuals in Mexico City, AMR ancestry was found to exert an additional influence beyond these factors, highlighting the importance of considering ancestry, alongside established risk factors such as BMI, when investigating the determinants of type 2 diabetes prevalence across different regions of the world.

### Contributors

### Data sharing

Data from the Mexico City Prospective Study are available to bona fide researchers. For more details, the study's Data and Sample Sharing policy is available in English or Spanish from https://www.ctsu.ox.ac.uk/research/mcps. Available study data can be examined in detail through the study's Data Showcase, available at https://datashare.ndph.ox.ac.uk/mexico/.

## Declaration of interests

JRE reports grants to the University of Oxford from AstraZeneca and Regeneron Pharmaceuticals. All other authors declare no competing interests.
