# Supplementary material for: The effect of Indigenous American genomic ancestry on type 2 diabetes in Mexico: an analysis of 134 548 individuals from the Mexico City Prospective Study
Source: Lancet Public Health. 2026 Jan 27;11(2):e111–9. doi: 10.1016/S2468-2667(25)00305-6 (PMC12873227; doi:10.1016/S2468-2667(25)00305-6)

THE LANCET
Public Health

Supplementary appendix

This appendix formed part of the original submission and has been peer reviewed. We post it as supplied by the authors.

Supplement to: Berumen J, Kuri-Morales P, Torres JM, et al. The effect of Indigenous American genomic ancestry on type 2 diabetes in Mexico: an analysis of 134 548 individuals from the Mexico City Prospective Study. *Lancet Public Health* 2026; **11:** e111–19.

The impact of Indigenous American ancestry on type 2 diabetes in Mexico: an analysis of 134,548 individuals from the Mexico City Prospective Study

Online Appendix, Table of Contents

Page

Webtables

1	Baseline characteristics of 90,688 included women by tenth of AMR proportion	2
2	Baseline characteristics of 43,860 included men by tenth of AMR proportion	3

Webfigures

1	Odds ratios for prediabetes and type 2 diabetes by age and level of adjustment	4
2	Impact of additional adjustment for a type 2 diabetes polygenic risk score (T2D-GRS)	5

Webtable 1. Baseline characteristics of 90,688 included women by tenth of AMR percentage

	Tenth of Indigenous American Ancestry Percentage									
	I	II	III	IV	V	VI	VII	VIII	IX	X
	(35%)	(47%)	(53%)	(58%)	(63%)	(69%)	(74%)	(81%)	(87%)	(95%)
Age, years	54.3±12.9	52.6±12.6	51.7±12.3	51.5±12.4	50.7±12.1	50.6±12.2	50.5±12.2	50.8±12.3	51.7±12.5	51.7±11.5
Adiposity										
BMI, kg/m^2	28.9±5.4	29.6±5.6	29.6±5.5	29.7±5.5	29.8±5.3	29.8±5.3	29.8±5.3	29.8±5.2	29.7±5	29.4±4.9
Waist circumference, cm	92.2±13.1	93.6±12.9	93.5±12.6	93.7±12.5	93.6±12.3	93.6±12.2	93.8±12.1	93.8±11.7	93.9±11.3	93.3±11
Hip circumference, cm	106.7±11.8	107.4±12	107.1±11.7	106.9±11.7	106.9±11.4	106.6±11.4	106.5±11.4	106.1±11.3	105.6±10.8	104.3±10.5
Waist-hip ratio	0.86±0.07	0.87±0.07	0.87±0.07	0.88±0.07	0.88±0.07	0.88±0.07	0.88±0.07	0.88±0.07	0.89±0.07	0.89±0.06
District										
Iztapalapa	4132 (47.1)	5173 (57.4)	5295 (58.8)	5668 (61.7)	5647 (62.3)	5912 (64.8)	6095 (66.9)	6377 (69.9)	6644 (73.1)	6563 (71.1)
Coyoacan	4636 (52.9)	3832 (42.6)	3711 (41.2)	3515 (38.3)	3415 (37.7)	3208 (35.2)	3013 (33.1)	2744 (30.1)	2443 (26.9)	2665 (28.9)
Income, pesos	1457±3279	1098±3033	990±2469	922±2044	908±2303	849±1935	788±1960	670±1515	613±1344	528±1153
Educational level										
University/high school	715 (8.2)	1037 (11.5)	1068 (11.9)	1136 (12.4)	1087 (12.0)	1211 (13.3)	1270 (13.9)	1468 (16.1)	1736 (19.1)	2687 (29.1)
Middle school	3606 (41.1)	4176 (46.4)	4306 (47.8)	4547 (49.5)	4510 (49.8)	4574 (50.2)	4756 (52.2)	4877 (53.5)	5094 (56.1)	4972 (53.9)
Elementary school	2424 (27.6)	2444 (27.1)	2445 (27.1)	2362 (25.7)	2459 (27.1)	2356 (25.8)	2246 (24.7)	2038 (22.3)	1703 (18.7)	1203 (13.0)
Other	2023 (23.1)	1348 (15.0)	1187 (13.2)	1138 (12.4)	1006 (11.1)	979 (10.7)	836 (9.2)	738 (8.1)	554 (6.1)	366 (4.0)
Tobacco use										
Never	4561 (52.0)	5101 (56.6)	5220 (58.0)	5387 (58.7)	5274 (58.2)	5568 (61.1)	5802 (63.7)	6143 (67.4)	6574 (72.3)	7156 (77.5)
Former	1834 (20.9)	1690 (18.8)	1672 (18.6)	1723 (18.8)	1710 (18.9)	1656 (18.2)	1576 (17.3)	1519 (16.7)	1291 (14.2)	1249 (13.5)
Current	2373 (27.1)	2214 (24.6)	2114 (23.5)	2073 (22.6)	2078 (22.9)	1896 (20.8)	1730 (19.0)	1459 (16.0)	1222 (13.4)	823 (8.9)
Alcohol use										
Never	2028 (23.1)	2227 (24.7)	2310 (25.6)	2276 (24.8)	2310 (25.5)	2418 (26.5)	2335 (25.6)	2532 (27.8)	2574 (28.3)	3004 (32.6)
Former	907 (10.3)	1045 (11.6)	1082 (12.0)	1133 (12.3)	1029 (11.4)	1150 (12.6)	1113 (12.2)	1080 (11.8)	1141 (12.6)	1171 (12.7)
Current	5833 (66.5)	5733 (63.7)	5614 (62.3)	5774 (62.9)	5723 (63.2)	5552 (60.9)	5660 (62.1)	5509 (60.4)	5372 (59.1)	5053 (54.8)
Regular leisure-time physical activity										
None	6330 (72.2)	6907 (76.7)	7104 (78.9)	7325 (79.8)	7349 (81.1)	7468 (81.9)	7581 (83.2)	7792 (85.4)	7799 (85.8)	8198 (88.8)
At least 1 day a week	2438 (27.8)	2098 (23.3)	1902 (21.1)	1858 (20.2)	1713 (18.9)	1652 (18.1)	1527 (16.8)	1329 (14.6)	1288 (14.2)	1030 (11.2)
Fruit and vegetable consumption										
Never	75 (0.9)	68 (0.8)	75 (0.8)	72 (0.8)	65 (0.7)	66 (0.7)	57 (0.6)	56 (0.6)	53 (0.6)	71 (0.8)
1-2 per week	981 (11.2)	1166 (12.9)	1295 (14.4)	1280 (13.9)	1351 (14.9)	1364 (15.0)	1422 (15.6)	1440 (15.8)	1521 (16.7)	1746 (18.9)
3-4 per week	1813 (20.7)	2197 (24.4)	2300 (25.5)	2475 (27.0)	2459 (27.1)	2564 (28.1)	2624 (28.8)	2758 (30.2)	2727 (30.0)	2870 (31.1)
5-7 per week	5899 (67.3)	5574 (61.9)	5336 (59.2)	5356 (58.3)	5187 (57.2)	5126 (56.2)	5005 (55.0)	4867 (53.4)	4786 (52.7)	4541 (49.2)
Fried food consumption										
Never	1383 (15.8)	1191 (13.2)	1168 (13.0)	1132 (12.3)	1061 (11.7)	1073 (11.8)	1031 (11.3)	1065 (11.7)	981 (10.8)	1014 (11.0)
1-2 per week	5040 (57.5)	5360 (59.5)	5282 (58.6)	5419 (59.0)	5368 (59.2)	5348 (58.6)	5462 (60.0)	5458 (59.8)	5491 (60.4)	5587 (60.5)
3-4 per week	1271 (14.5)	1429 (15.9)	1496 (16.6)	1514 (16.5)	1551 (17.1)	1609 (17.6)	1595 (17.5)	1644 (18.0)	1611 (17.7)	1532 (16.6)
5-7 per week	1074 (12.2)	1025 (11.4)	1060 (11.8)	1118 (12.2)	1082 (11.9)	1090 (12.0)	1020 (11.2)	954 (10.5)	1004 (11.0)	1095 (11.9)
Diabetes										
No prediabetes or diabetes	6032 (68.8)	5687 (63.2)	5642 (62.6)	5658 (61.6)	5414 (59.7)	5394 (59.1)	5221 (57.3)	4978 (54.6)	4558 (50.2)	4522 (49.0)
Pre-diabetes	1610 (18.4)	1966 (21.8)	1952 (21.7)	1920 (20.9)	2005 (22.1)	1985 (21.8)	2113 (23.2)	2196 (24.1)	2392 (26.3)	2524 (27.4)
T2D	1126 (12.8)	1352 (15)	1412 (15.7)	1605 (17.5)	1643 (18.1)	1741 (19.1)	1774 (19.5)	1947 (21.3)	2137 (23.5)	2182 (23.6)
HbA1c, %										
In those without diabetes	5.4±0.4	5.4±0.4	5.4±0.4	5.4±0.4	5.4±0.4	5.5±0.4	5.5±0.4	5.5±0.4	5.5±0.4	5.5±0.4
In those with diabetes	8.3±2.1	8.6±2.3	8.6±2.3	8.7±2.3	8.7±2.3	8.8±2.3	8.9±2.4	8.9±2.4	8.9±2.4	9.1±2.5
In all participants	5.8±1.3	5.9±1.5	6±1.5	6±1.6	6.1±1.7	6.1±1.7	6.2±1.8	6.3±1.8	6.3±1.9	6.4±2
Amerindian ancestry										
AMR, proportion	0.349±0.078	0.470±0.021	0.532±0.016	0.585±0.015	0.635±0.015	0.687±0.015	0.743±0.017	0.805±0.019	0.872±0.019	0.947±0.027
EUR, proportion	0.575±0.095	0.460±0.043	0.405±0.032	0.358±0.029	0.314±0.027	0.269±0.024	0.221±0.022	0.166±0.021	0.108±0.020	0.043±0.024
AFR, proportion	0.062±0.038	0.059±0.033	0.053±0.025	0.048±0.023	0.043±0.021	0.037±0.018	0.030±0.015	0.022±0.012	0.015±0.009	0.006±0.006
EAS, proportion	0.015±0.040	0.011±0.019	0.010±0.012	0.009±0.011	0.008±0.009	0.008±0.008	0.007±0.007	0.007±0.007	0.006±0.006	0.004±0.005

Numbers shown are n (%) or mean ±SD. BMI=Body mass index; HbA1c=Glycosylated haemoglobin; AMR=Amerindian ancestry; EUR=European ancestry; AFR=African ancestry; EAS=East Asian ancestry; T2D=Type 2 diabetes. Prediabetes defined as no previous diagnosis of diabetes and HbA1c in the range ≥5.7 to <6.5%.

* Nearly two-thirds of the women reported not having a salary (75% of them were married or living with a partner).

Webtable 2. Baseline characteristics of 43,860 included men by tenth of AMR percentage

	Tenth of Indigenous American Ancestry Percentage									
	I	II	III	IV	V	VI	VII	VIII	IX	X
	(35%)	(47%)	(53%)	(58%)	(63%)	(69%)	(74%)	(81%)	(87%)	(95%)
Age, years	56.2±13.1	54.4±13	52.8±12.6	52.4±12.6	52.4±12.6	51.6±12.4	51.4±12.7	51.5±12.5	52.8±13.2	53.3±12.9
Adiposity										
BMI, kg/m^2	27.4±4.2	27.7±4.5	27.8±4.4	27.9±4.2	28±4.3	28.2±4.6	28.2±4.5	28.3±4.4	28.2±4.1	28±4.2
Waist circumference, cm	97.3±11.4	97.2±11.1	96.8±10.8	96.6±10.7	96.5±10.2	96.6±10.7	96.3±10.3	96.3±10.4	96.1±10.4	95.1±9.7
Hip circumference, cm	102±8.6	101.8±8.6	101.7±8.3	101.4±8.2	101.3±8.2	101.3±8	100.9±8.3	100.7±8.2	100.3±7.6	99.5±7.5
Waist-hip ratio	0.95±0.07	0.95±0.07	0.95±0.06	0.95±0.06	0.95±0.06	0.95±0.07	0.95±0.07	0.96±0.07	0.96±0.07	0.96±0.06
District										
Iztapalapa	2046 (44.4)	2397 (54.2)	2387 (53.4)	2455 (56.8)	2574 (58.5)	2594 (59.7)	2757 (62.6)	2809 (64.4)	3036 (70)	2818 (67.2)
Coyoacan	2562 (55.6)	2028 (45.8)	2079 (46.6)	1864 (43.2)	1823 (41.5)	1754 (40.3)	1648 (37.4)	1553 (35.6)	1301 (30)	1375 (32.8)
Income, pesos	5018±8080	3973±6951	3659±5119	3424±4871	3219±4479	3040±3784	2977±4106	2727±3502	2547±3933	2354±4616
Educational level										
University/high school	302 (6.6)	402 (9.1)	370 (8.3)	350 (8.1)	362 (8.2)	392 (9)	367 (8.3)	356 (8.2)	439 (10.1)	607 (14.5)
Middle school	1369 (29.7)	1568 (35.4)	1643 (36.8)	1672 (38.7)	1866 (42.4)	1810 (41.6)	1914 (43.5)	2066 (47.4)	2151 (49.6)	2254 (53.8)
Elementary school	1074 (23.3)	1163 (26.3)	1211 (27.1)	1248 (28.9)	1204 (27.4)	1199 (27.6)	1234 (28)	1138 (26.1)	1075 (24.8)	861 (20.5)
Other	1863 (40.4)	1292 (29.2)	1242 (27.8)	1049 (24.3)	965 (21.9)	947 (21.8)	890 (20.2)	802 (18.4)	672 (15.5)	471 (11.2)
Tobacco use										
Never	849 (18.4)	857 (19.4)	848 (19)	841 (19.5)	844 (19.2)	838 (19.3)	869 (19.7)	901 (20.7)	933 (21.5)	1093 (26.1)
Former	1774 (38.5)	1584 (35.8)	1564 (35)	1526 (35.3)	1534 (34.9)	1550 (35.6)	1577 (35.8)	1593 (36.5)	1590 (36.7)	1650 (39.4)
Current	1985 (43.1)	1984 (44.8)	2054 (46)	1952 (45.2)	2019 (45.9)	1960 (45.1)	1959 (44.5)	1868 (42.8)	1814 (41.8)	1450 (34.6)
Alcohol use										
Never	334 (7.2)	302 (6.8)	261 (5.8)	270 (6.3)	288 (6.5)	268 (6.2)	241 (5.5)	264 (6.1)	256 (5.9)	260 (6.2)
Former	707 (15.3)	721 (16.3)	783 (17.5)	787 (18.2)	792 (18)	804 (18.5)	804 (18.3)	793 (18.2)	815 (18.8)	856 (20.4)
Current	3567 (77.4)	3402 (76.9)	3422 (76.6)	3262 (75.5)	3317 (75.4)	3276 (75.3)	3360 (76.3)	3305 (75.8)	3266 (75.3)	3077 (73.4)
Regular leisure-time physical activity										
None	2972 (64.5)	2993 (67.6)	3022 (67.7)	2996 (69.4)	3108 (70.7)	3056 (70.3)	3110 (70.6)	3203 (73.4)	3119 (71.9)	3204 (76.4)
At least 1 day a week	1636 (35.5)	1432 (32.4)	1444 (32.3)	1323 (30.6)	1289 (29.3)	1292 (29.7)	1295 (29.4)	1159 (26.6)	1218 (28.1)	989 (23.6)
Fruit and vegetable consumption										
Never	83 (1.8)	99 (2.2)	87 (1.9)	78 (1.8)	69 (1.6)	79 (1.8)	84 (1.9)	77 (1.8)	58 (1.3)	56 (1.3)
1-2 per week	849 (18.4)	883 (20)	938 (21)	891 (20.6)	993 (22.6)	965 (22.2)	1039 (23.6)	993 (22.8)	960 (22.1)	989 (23.6)
3-4 per week	1107 (24)	1216 (27.5)	1277 (28.6)	1280 (29.6)	1308 (29.7)	1272 (29.3)	1382 (31.4)	1367 (31.3)	1468 (33.8)	1451 (34.6)
5-7 per week	2569 (55.8)	2227 (50.3)	2164 (48.5)	2070 (47.9)	2027 (46.1)	2032 (46.7)	1900 (43.1)	1925 (44.1)	1851 (42.7)	1697 (40.5)
Fried food consumption										
Never	605 (13.1)	475 (10.7)	439 (9.8)	477 (11)	435 (9.9)	428 (9.8)	461 (10.5)	426 (9.8)	403 (9.3)	446 (10.6)
1-2 per week	2234 (48.5)	2221 (50.2)	2225 (49.8)	2113 (48.9)	2236 (50.9)	2188 (50.3)	2220 (50.4)	2223 (51)	2272 (52.4)	2152 (51.3)
3-4 per week	873 (18.9)	857 (19.4)	894 (20)	920 (21.3)	898 (20.4)	945 (21.7)	877 (19.9)	930 (21.3)	897 (20.7)	892 (21.3)
5-7 per week	896 (19.4)	872 (19.7)	908 (20.3)	809 (18.7)	828 (18.8)	787 (18.1)	847 (19.2)	783 (18)	765 (17.6)	703 (16.8)
Diabetes										
No prediabetes or diabetes	2966 (64.4)	2787 (63)	2790 (62.5)	2696 (62.4)	2645 (60.2)	2630 (60.5)	2583 (58.6)	2488 (57)	2366 (54.6)	2228 (53.1)
Pre-diabetes	963 (20.9)	930 (21)	933 (20.9)	832 (19.3)	938 (21.3)	931 (21.4)	959 (21.8)	890 (20.4)	967 (22.3)	1005 (24)
T2D	679 (14.7)	708 (16)	743 (16.6)	791 (18.3)	814 (18.5)	787 (18.1)	863 (19.6)	984 (22.6)	1004 (23.1)	960 (22.9)
HbA1c, %										
In those without diabetes	5.4±0.4	5.4±0.4	5.4±0.4	5.4±0.4	5.5±0.4	5.5±0.4	5.5±0.4	5.5±0.4	5.5±0.4	5.5±0.4
In those with diabetes	8.4±2.2	8.6±2.3	8.6±2.4	8.7±2.5	8.8±2.3	8.8±2.3	8.7±2.4	8.9±2.4	8.8±2.3	8.9±2.6
In all participants	5.9±1.4	6±1.5	6±1.6	6±1.7	6.1±1.7	6.1±1.7	6.1±1.7	6.2±1.9	6.3±1.8	6.3±1.9
Amerindian ancestry										
AMR, proportion	0.347±0.079	0.47±0.021	0.532±0.016	0.585±0.014	0.635±0.015	0.687±0.015	0.743±0.017	0.805±0.019	0.872±0.019	0.946±0.027
EUR, proportion	0.577±0.099	0.461±0.042	0.406±0.033	0.360±0.027	0.315±0.025	0.269±0.023	0.220±0.022	0.167±0.021	0.108±0.020	0.044±0.024
AFR, proportion	0.060±0.038	0.058±0.031	0.052±0.025	0.047±0.022	0.042±0.019	0.036±0.017	0.029±0.015	0.022±0.011	0.014±0.009	0.006±0.006
EAS, proportion	0.017±0.050	0.011±0.021	0.010±0.015	0.009±0.011	0.008±0.010	0.008±0.009	0.007±0.007	0.007±0.007	0.006±0.006	0.004±0.005

Numbers shown are n (%) or mean ±SD. BMI=Body mass index; HbA1c=Glycosylated haemoglobin; AMR=Amerindian ancestry; EUR=European ancestry; AFR=African ancestry; EAS=East Asian ancestry; T2D=Type 2 diabetes. Prediabetes defined as no previous diagnosis of diabetes and HbA1c in the range ≥5.7 to <6.5%.

* Nearly two-thirds of the women reported not having a salary (75% of them were married or living with a partner).

Webfigure 1. Odds ratios for prediabetes and type 2 diabetes by age and level of adjustment. Minimal-adjustment includes adjustment for age and sex. Full-adjustment also includes socio-economic factors, lifestyle factors, body mass index, and waist-hip ratio. Horizontal lines through points reflect 95% confidence intervals. CI= Confidence interval, OR=Odds ratio.

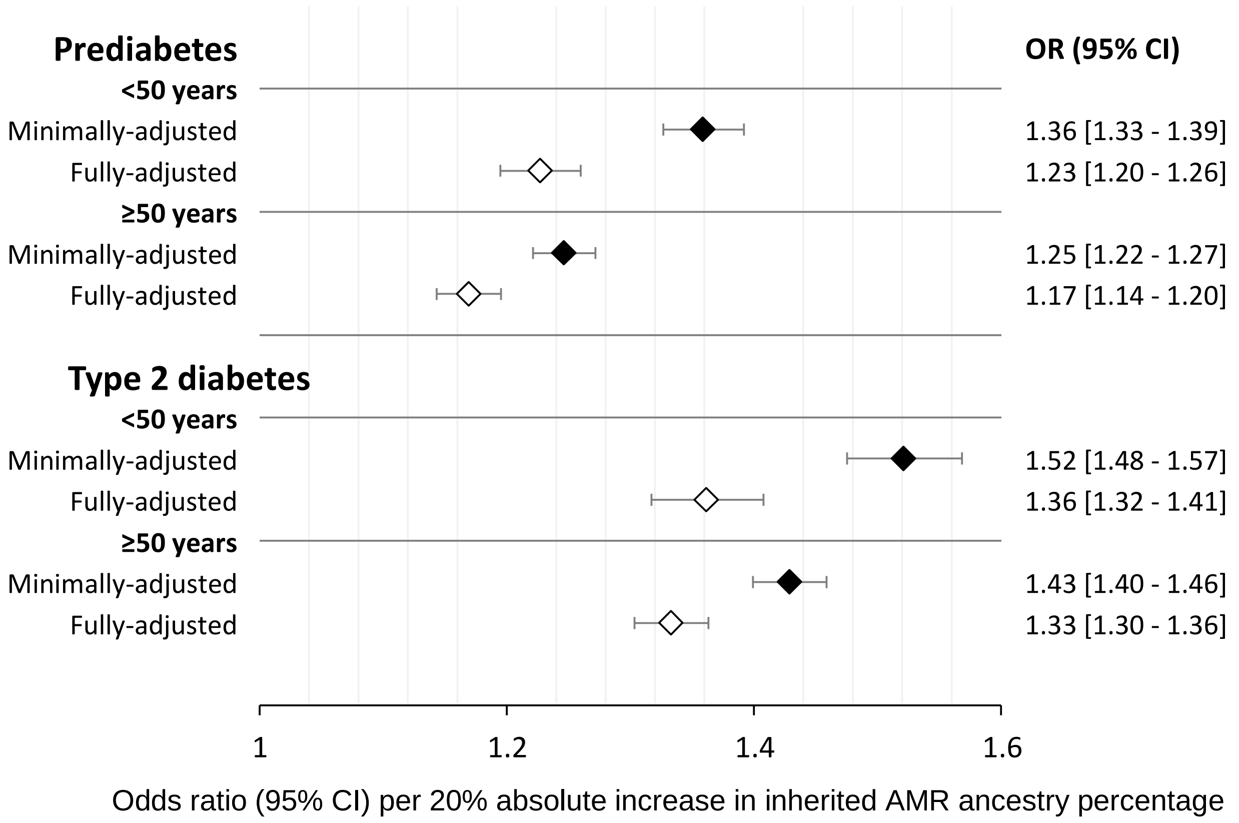

Webfigure 2. Impact of *additional* adjustment for a type 2 diabetes polygenic risk score (T2D-GRS). Panel A shows boxplots of a T2D genetic risk score (GRS) by tenths of the distribution of the AMR ancestry proportion. The GRS was constructed using weights from a trans-ancestry T2D GWAS meta-analysis reported by the T2D Global Genomics Initiative. Panel B repeats the main results from Figure 3 (minimal and full adjustment), but also includes (as black diamonds) *further* adjustment for this T2D-GRS. CI= Confidence interval, GRS=genetic risk score, OR=Odds ratio.

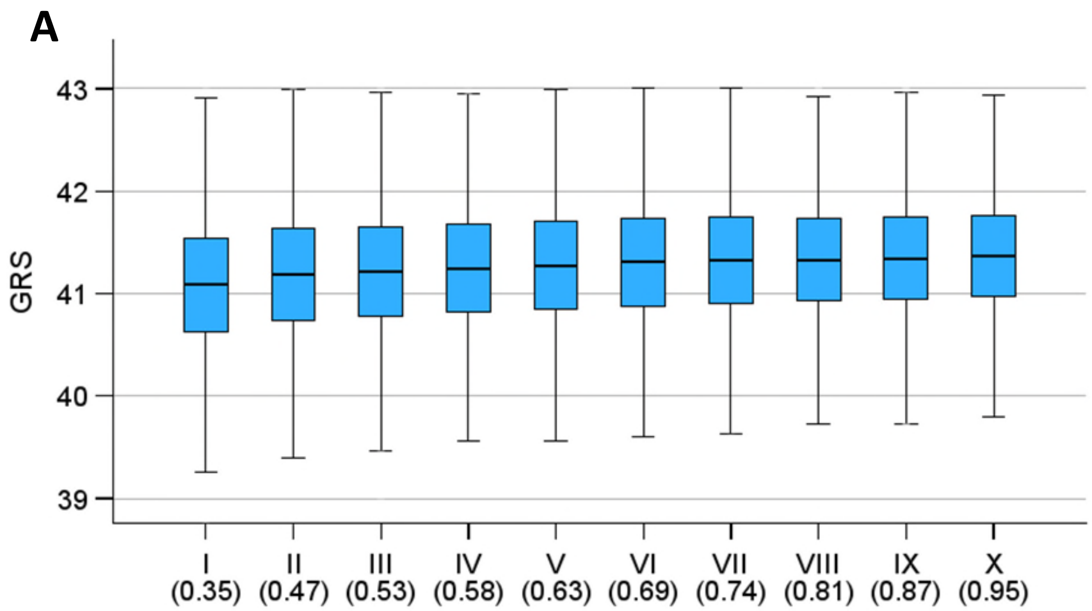

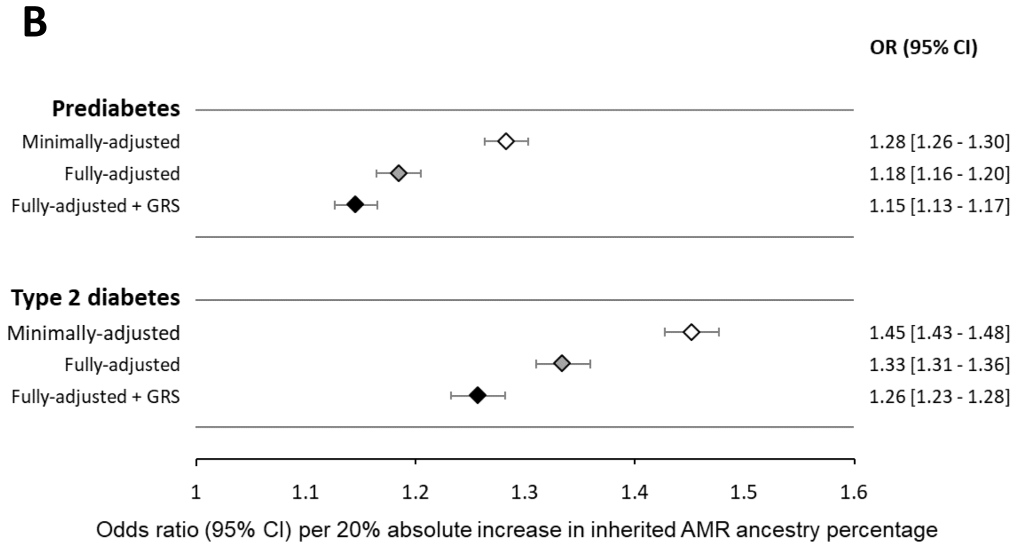

Supplement: Supplementary appendix [file mmc1.pdf]
